# Magnetic Resonance Left Ventricle Mass-Index/Fibrosis: Long-Term Predictors for Ventricular Arrhythmia in Hypertrophic Cardiomyopathy—A Retrospective Registry

**DOI:** 10.3390/jcdd10030120

**Published:** 2023-03-13

**Authors:** Habib Rehman Khan, Philip Rodwell, Ahmed Hasan Taha, Ahmed Goha, Mobeen Ahmed, Andrew Peter Thain, Konstantinos Somarakis, Ayman Al-Atta, Bara Erhayiem, Akhlaque Uddin, Thomas Mathew

**Affiliations:** 1 Department of Cardiology, Nottingham University NHS Trust, Hucknall Road, Nottingham NG51PB, UK; 2 London Health Sciences Centre, University of Western Ontario, Windermere Road, London, ON N6G5A5, Canada; ahmed.goha@lhsc.on.ca (A.G.);; 3 Cardiology Department, Tanta University, Al-Geish Street, Tanta 31512, Egypt

**Keywords:** hypertrophic cardiomyopathy (HCM), implantable cardioverter defibrillation (ICD), left ventricular mass, risk stratification, left ventricular wall thickness, late gadolinium enhancement (LGE), ventricular arrhythmia (VA)

## Abstract

*Objective*: We aimed to study the long-term association of LV mass index (LV_MI_) and myocardial fibrosis with ventricular arrhythmia (VA) in a population of patients with confirmed hypertrophic cardiomyopathy (HCM) using cardiac magnetic resonance imaging (CMR). *Methods*: We retrospectively analyzed the data in consecutive HCM patients confirmed on CMR referred to an HCM clinic between January 2008 and October 2018. Patients were followed up yearly following diagnosis. Baseline demographics, risk factors and clinical outcomes from cardiac monitoring and an implanted cardioverter defibrillator (ICD) were analyzed for association of LV_MI_ and LV late gadolinium enhancement (LV_LGE_) with VA. Patients were then allocated to one of two groups according to the presence of VA (Group A) or absence of VA (Group B) during the follow-up period. The transthoracic echocardiogram (TTE) and CMR parameters were compared between the two groups. *Results*: A total of 247 patients with confirmed HCM (age 56.2 ± 16.6, male = 71%) were studied over the follow-up period of 7 ± 3.3 years (95% CI = 6.6–7.4 years). LV_MI_ derived from CMR was higher in Group A (91.1 ± 28.1 g/m^2^ vs. 78.8 ± 28.3 g/m^2^, *p* = 0.003) when compared to Group B. LV_LGE_ was higher in Group A (7.3 ± 6.3% vs. 4.7 ± 4.3%, *p* = 0.001) when compared to Group B. Multivariable Cox regression analysis showed LV_MI_ (hazard ratio (HR) = 1.02, 95% CI = 1.001–1.03, *p* = 0.03) and LV_LGE_ (HR = 1.04, 95% CI = 1.001–1.08, *p* = 0.04) to be independent predictors for VA. Receiver operative curves showed higher LV_MI_ and LV_LGE_ with a cut-off of 85 g/m^2^ and 6%, respectively, to be associated with VA. *Conclusions*: LV_MI_ and LV_LGE_ are strongly associated with VA over long-term follow-up. LV_MI_ requires more thorough studies to consider it as a risk stratification tool in patients with HCM.

## 1. Introduction

Hypertrophic cardiomyopathy (HCM) is the most common inherited cardiac condition with an incidence of 1 in 500 births, characterized by cardiac hypertrophy, usually asymmetrical with the greatest involvement of the basal interventricular septum, with wall thickness ≥ 15 mm in adults or ≥13 mm without higher loading conditions [1,2,3,4,5,6]. Several HCM phenotypes have been identified and linked to >1400 mutations in 11 sarcomere protein genes with heterogenous presentation, diverse pathophysiology, and variable course [7].

HCM may present with symptoms of exertional fatigue, dyspnea, syncope, sudden cardiac arrest (SCA), or sudden cardiac death (SCD), or incidentally identified during routine screening with abnormal electrocardiogram [2,8,9,10].

HCM is classified according to morphological appearance or genetic phenotype [11]. Initially, two distinct phenotypic patterns were described: early onset in younger individuals with very aggressive features of obstruction and ventricular arrhythmias (VA); or less aggressive late onset during adulthood [1].

Over the last few decades, the development of imaging modalities has significantly changed our understanding of HCM phenotypes, disease progression and management [2,3,5,9]. The target for HCM management is symptom relief either by reducing the ventricular rate to improve cardiac filling or reducing left ventricular outflow tract (LVOT) obstruction by medication or septal myomectomy. Identification of high-risk features is crucial for the prevention of SCD and the consideration of an implantable cardioverter defibrillator (ICD) [1,9].

HCM is recognized as the major cause of mortality among young individuals. The annual incidence of sudden cardiac death (SCD) is 0.5–1%. The percentage of SCD is higher among younger patients with HCM and decreases with aging. Current guidelines endorse an implantable cardioverter defibrillator (ICD) for secondary prevention and for primary prevention of SCD in high-risk individuals with HCM [12].

SCD has been the most deleterious complication of HCM for more than half a century, mainly in those of a young age. The implementation of ICDs over the last two decades for both secondary and primary prevention of high-risk HCM patients significantly ameliorated the outcome of HCM patients. Several risk schemes have been developed to identify high-risk patients in need for primary ICD employment. However, controversy remains for the optimal risk stratification scheme to identify high-risk patients [13].

The American Heart Association (AHA), the American College of Cardiology (ACC) and the European Society of Cardiology (ESC) developed risk stratification models to improve the prediction of SCD with the inclusion of more risk factors [1,14]. Multiple studies showed the limitation of guidelines to properly differentiate low-risk from high-risk individuals, which may leave some individuals unprotected without an ICD [15,16]. Recognizing high-risk individuals is challenging due to the variable phenotype of HCM missed on TTE, resulting in lower predictive value of the risk factors, as well as low clinical event rates [17]. CMR provides a better assessment of the left ventricular wall thickness (LV_WT_), LV mass index (LV_MI_) and scar quantifying using left ventricle late gadolinium enhancement (LV_LGE_), which has shown to be a positive predictor of SCD [18,19,20]. LV_MI_ is currently not described as a risk factor for SCD in patients with HCM [14]. Further larger studies are needed to improve risk stratification schemes by integration of novel CMR markers and avoid inappropriate ICD implementation [21].

HCM patients report a higher incidence of arrhythmias, including atrial fibrillation, ventricular premature beats (VPB), non-sustained ventricular tachycardia (NSVT), sustained ventricular tachycardia (SVT), ventricular fibrillation (VF), heart failure and SCD compared to the general population of a similar age [12,22]. Multiple studies showed a significant correlation between NSVT and the risk of SCD in patients with HCM. However, the consideration of NSVT as a risk marker is still controversial [23].

We aim to retrospectively study the impact of LV_MI_ and LV_LGE_ in HCM patients.

## 2. Objectives

The main objectives of this retrospective study are:

- To evaluate the association of LV_MI_ and LV_LGE_ with VT.
- To compare differences in the ESC risk score using TTE and CMR.

## 3. Methods

Our study is a retrospective longitudinal observational study that included consecutive patients referred to outpatient HCM clinics of a single tertiary center with HCM diagnosed/confirmed diagnosis on CMR and followed between 2008 and 2018.

Local ethics committee approval was obtained from the Research and Development office of Nottingham University Hospitals NHS Trust, ID20-148C. The study was designed in 2018 and, at the time of conceptualization and design, no patient or public involvement was required or obtained for this retrospective study.

Baseline demographics and risk factors were retrieved from completed clinical records. HCM diagnostic criteria was LV_WT_ ≥ 15 mm in adults or ≥13 mm in patients with a genetic mutation, after exclusion of secondary causes [3,4,11]. Initial and follow-up echocardiograms were performed in our tertiary center by an accredited BSE echo sonographer and reported by a level 3 cardiologist. LV_WT_ was measured on TTE in the parasternal long- and short-axis views at end diastole using a standard calibration scale [24]. ICDs were implanted as part of primary prevention guided by AHA and ESC guidelines before 2014 and the ESC risk score after 2014 [2,3,14]. Yearly follow-up for patients with echocardiogram and 48 h Holter monitoring occurred at each visit. LV ESC risk scores were calculated retrospectively for all patients using TTE and CMR, and categorized into low (<4%), moderate (4–6%) or high risk (≥6%) for SCD.

For primary outcome, patients were divided into two groups:

Group A: patients with an incidence of VA:

- NSVT: ≥3 consecutive ventricular beats ≥120 beats per minute captured on ECG, Holter monitoring or cardiac implantable electronic device (CIED) during follow-up.
- Therapy: Appropriate anti-tachycardia pacing (ATP) or cardioversion/defibrillation provided by ICD or external cardioversion for sustained ventricular tachycardia (VT) or ventricular fibrillation (VF).

Group B: patients who had no VA detected during the follow-up and no ICD therapies.

## 4. Cardiac MRI

CMR examinations were performed using 1.5T scanners (Philips ACS-NT 1.5 T Gyroscan-Intera, Best, The Netherlands or Siemens Sonata 1.5 T, Erlangen, Germany) and a commercial cardiac coil. Electrocardiographic-gated, steady-state, free breath-hold sequential cines of 10 mm short-axis slices were acquired starting parallel to the atrioventricular ring and covering the entire ventricle. Late gadolinium enhancement images were acquired 15 min after the intravenous administration of 0.2 mmol/kg of gadolinium-DTPA (Magnevist, Schering; Berlin, Germany). A 2D segmented inversion-recovery sequence with breath-hold was acquired in the same views as the cine images.

**Figure 1:**
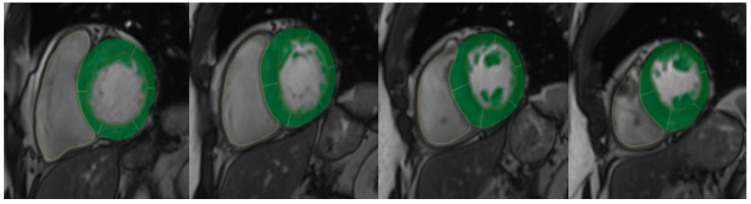
LV mass measurement on short-axis using CMR.

**Figure 2:**
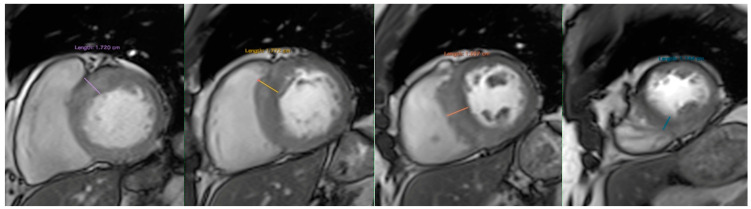
LV wall thickness measurement on CMR.

**Figure 3:**
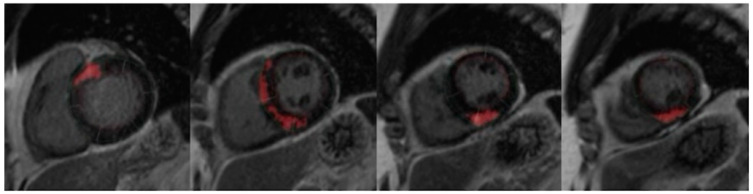
CMR calculation of late gadolinium enhancement of the left ventricle using 6-SD threshold.

LV volume, ejection fraction, mass and myocardial fibrosis were measured using standard volumetric and semi-automated techniques with commercially available software (Qmass MR version 6 1.6, Medis Medical Imaging Systems, The Netherlands), as shown in Figure 1. LV contours were outlined according to SCMR guidelines [25]. Trabeculae and papillary muscles were obviated from LV mass calculation [26]. LV_WT_ was defined as the greatest dimension at any site within the LV wall (Figure 2). To assess myocardial fibrosis (LV_LGE_), all short-axis slices from base to apex were inspected visually to compare with areas of normal myocardium. Myocardial fibrosis was quantified at a grey-scale threshold of six standard deviations (SDs) above the mean signal intensity for normal myocardium (Figure 3). The quantity of LV_LGE_ was expressed as a percentage of the total LV myocardial mass [27]. The LV_LGE_ analysis was performed on anonymized datasets twice by two experienced readers. Any discrepancies in analysis between the two readers were then adjudicated by a senior observer. To assess interobserver variability for the extent of LV_LGE_, 100 randomly selected studies were reanalyzed by the second reader.

## 5. Statistics

All parametric continuous values were statistically analyzed using Student’s *t*-test and presented as mean and SD. Non-parametric continuous values were analyzed using the Mann–Whitney test and presented as median and interquartile ranges. All categorical values were statistically analyzed using chi-square test or Pearson–Spearman test. Univariable Cox regression hazard proportional analysis was performed to evaluate hazard ratios (HR) and 95% confidence intervals (CI). Univariable results with *p* value < 0.1 were used in multivariable Cox regression analysis to establish if there was independent predictor of VA as defined in the methodology. Variance inflation factor was used to detect collinearity.

Receiver operative characteristic (ROC) curve analysis was used to define optimal cut-off values for LV_MI_ and LV_LGE_ as a test to predict VA. Negative and positive predictive values (NPV and PPV) were calculated for LV_MI_ and LV_LGE_. These cut-off values were applied to measure incidence of VT using Kaplan–Meier curves measured using log rank test and HR. Correlation between LV_MI_ and LV_LGE_ was measured using linear regression and Bland–Altman plots. Statistical *p* values < 0.05 were considered significant. SPSS version 25 or higher (IBM corporation, Armonk, New York, NY, USA) were used for statistical analysis.

## 6. Results

Our study included data of 252 patients (56.2 ± 16.3 years, males 69.0%) with confirmed HCM, and were followed up for an average of 6.6 ± 3.3 years. Baseline demographics and imaging measurements of all the subjects are listed in Table 1. Patients had TTE within 3 months prior to CMR. There were no differences between Group A and B with regard to SCD family history (23.2% vs. 25.8%, *p* = 0.75), and death from all causes during follow-up (15.9% vs. 9.7%, *p* = 0.18).

Patients with increased ESC risk had an ICD inserted (35 patients): 19 patients before 2014 and 16 patients after the 2014 guidelines. Twenty-four patients (38.1%) of Group A had ICD insertion compared to eleven patients (6.0%) of Group B, *p* < 0.01. SCD was aborted in 7 out of the 24 ICD patients in Group A by therapies delivered by ICD (*n* = 5) and external defibrillation (*n* = 2).

CMR-measured LV_WT_ was higher compared to TTE in all patients (19.5 ± 5.2 mm vs. 16.7 ± 5.6 mm, *p* < 0.001). CMR LV_WT_ was significantly higher in Group A compared to Group B (20.9 ± 7.0 mm vs. 19.0 ± 4.4 mm, *p* = 0.01). CMR-measured LV_MI_ was significantly higher in Group A compared to Group B (90.3 ± 27.3 g/m^2^ vs. 78.7 ± 28.3 g/m^2^, *p* = 0.004), Additionally, LV_LGE_ was also higher in Group A (9.2 ± 7.9% vs. 6.2 ± 5.9%, *p* = 0.02), as shown in Table 1.

### 6.1. Correlation of LV_WT_ and LV_MI_ with LV Fibrosis

**Figure 4:**
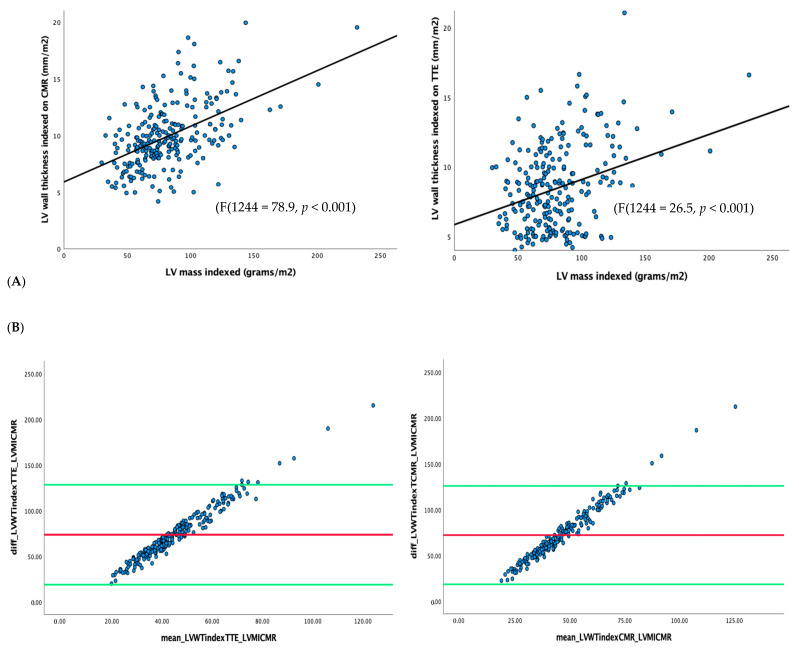
Correlation between LV mass measured from CMR and LV maximum thickness derived from CMR (Panel (**A**)) and TTE (Panel (**B**)). Bland–Altman plot: (**A**): mean = 73.5, 95% CI = 18.7–128.2, *p* < 0.001. (**B**): mean = 72.0, 95% CI = 18.3–125.8, *p* < 0.001.

**Figure 5:**
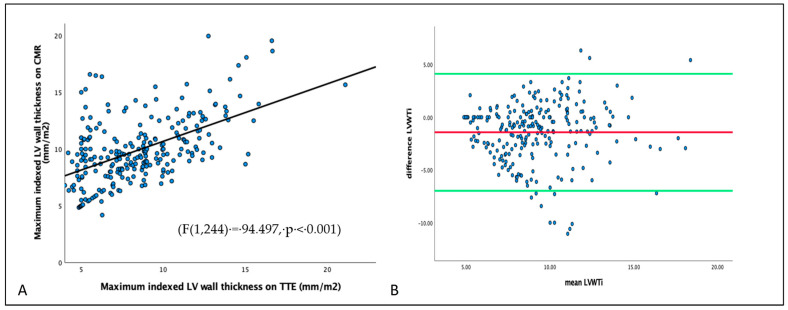
Panel (**A**): Correlation between LV_WT_ measured between CMR and TTE. Panel (**B**)*:* Bland–Altman plot of indexed LV_WT_ correlation between TTE and CMR. Mean = −1.43, 95% CI = −6.97–4.12, *p* = 0.44.

**Figure 6:**
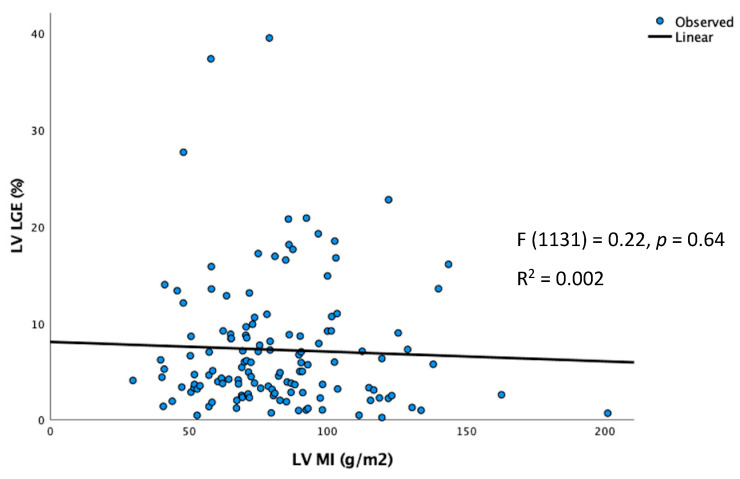
Correlation between LV_MI_ and LV_LGE_ measured using CMR.

LV_MI_ had a weak correlation with LV_WT_ derived from CMR (R^2^ = 0.245) and TTE (R^2^ = 0.098) (Figure 4). LV_WT_ was weakly correlated between CMR and TTE (R^2^ = 0.279) (Figure 5). LV_MI_ was poorly correlated with LV_LGE_ (R^2^ = 0.002) (Figure 6).

### 6.2. Predictors of VT

Cox regression univariable and multivariable analyses were performed to identify predictors for VA prior to the event in the HCM population, as shown in Table 2.

Univariable Cox regression analysis shows TTE LV_WT_ (HR= 1.03, 95% CI = 0.99–1.09, *p* = 0.18), CMR LV_MI_ (HR = 1.01, 95% CI = 1.004–1.02, *p* = 0.006) and LV_LGE_ (HR = 1.07, 95% CI = 1.01–1.13, *p* = 0.03) were associated with VT. Multivariable Cox regression analysis showed CMR LV_MI_ (HR = 1.02, 95% CI= 1.01–1.04, *p* = 0.003) and CMR LV_LGE_ (HR = 1.08, 95% CI = 1.02–1.15, *p* = 0.007) to be the only independent predictors of VT.

**Figure 7:**
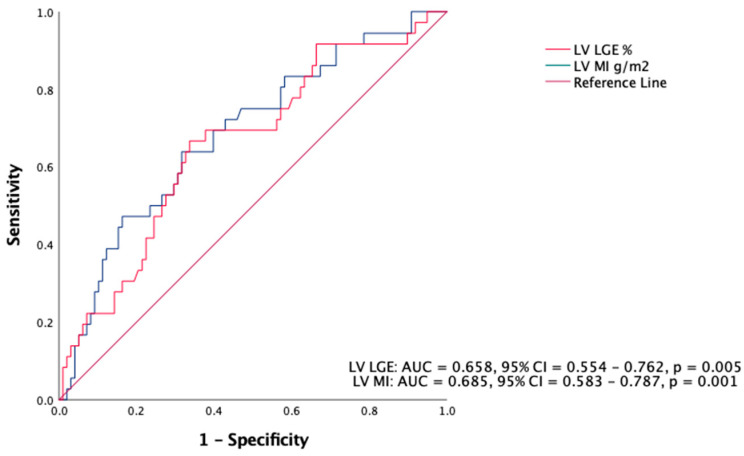
ROC curve analysis of LV_MI_ and LV_LGE_ in predicting ventricular arrhythmia.

ROC curve analysis showed LV_MI_ (AUC= 0.638, 95% CI = 0.561–0.714, *p* = 0.001) and LV_LGE_ (AUC= 0.633, 95% CI = 0.530–0.736, *p* = 0.015) to be good markers for VT (Figure 7). A cut-off value for LV_MI_ of >85.0 g/m^2^ (sensitivity = 64%, specificity = 68%, NPV = 0.81, PPV = 0.35) and for LV_LGE_ >6% (sensitivity = 66.7%, specificity = 66.3%, NPV = 0.84, PPV = 0.42) were used in Kaplan–Meier analysis to evaluate freedom from VT.

**Figure 8:**
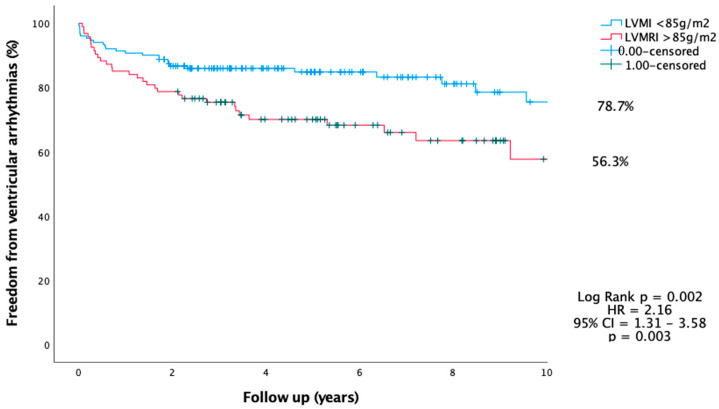
Kaplan–Meier analysis of LV mass and freedom from ventricular arrhythmia over a follow-up of 10 years.

Kaplan–Meier analysis showed higher freedom from VA in patients with LV_MI_ < 85.0 g/m^2^ compared to LV_MI_ > 85 g/m^2^ (78.7% vs. 56.3%, *p* =0.003, HR 2.16, 95% CI = 1.31–3.58, *p* = 0.003) (Figure 8).

**Figure 9:**
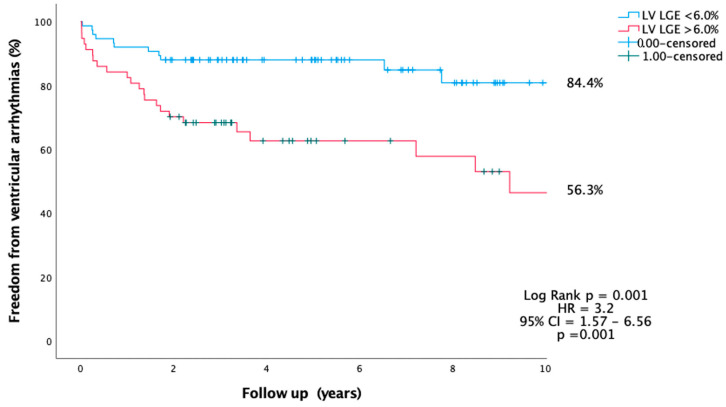
Kaplan Meier analysis of LV_LGE_ and freedom from ventricular arrhythmia over a follow-up of 10 years.

Similarly, there was significantly higher freedom from VA in patients with LV_LGE_ < 6% compared to LV_LGE_ > 6% (84.4% vs. 56.3%, *p* = 0.001, HR = 3.2, 95% CI = 1.57–6.56, *p* = 0.001) (Figure 9).

## 7. Discussion

Our study is one of the longest retrospective studies to observe HCM patients, and the main findings are as follows:

1. Higher LV_MI_ is associated with VA and can be considered for risk stratification of SCD in HCM.
2. LV_MI_ > 85 g/m^2^ and LV_LGE_ > 6% are associated with VA.
3. LV_WT_ using TTE or CMR was only weakly correlated with LV_MI_ on CMR.
4. LV_MI_ and LV_LGE_ were independent predictors of VA during follow-up.

CMR has superior spatial resolution and provides a multidimensional reconstruction of the heart. However, current guidelines are still considering CMR only for elucidation of diagnosis when there is a diagnostic dilemma, and do not include CMR for risk stratification for patients with HCM [13].

**HCM risk score using TTE and CMR**

American guidelines identified LV_WT_ > 30 mm as a major risk factor for SCD and LV_WT_ measured by TTE was incorporated into the European HCM Risk-SCD calculator [28]. TTE is the most common imaging modality used for diagnosis and risk stratification of HCM patients. However, LV_MI_ and LV_WT_ assessments are limited by asymmetric distribution of hypertrophy, echo window limitations resulting in underestimating or overestimating maximum LV_WT_, the inclusion of papillary muscles and right ventricular insertion into LV [19,29,30,31]. Several studies reported considerable variation of LV_WT_ assessed with TTE vs. MRI. Śpiewak et al. developed a simulation model comparing LV_WT_ measured by TTE vs. CMR for risk stratification according to the European HCM Risk-SCD calculator. The discrepancy for which CMR measured LV_WT_ translated to significant differences in the five-year risk of SCD [28].

CMR allows earlier and accurate diagnosis of HCM with early detection of myocardial fibrosis [9,14,30,31]. CMR manifests superior accuracy and reproducibility of LV_WT_ and LV_MI_ assessment compared to TTE [28,32,33], particularly when TTE imaging of LV is inadequate as supported by the British Society of Echocardiography and the current guidelines [3,9,14,30].

Another study showed the valuable role of using CMR in risk stratification of HCM patients. A study by Freitas et al., 2019, conducted a multicenter retrospective analysis of HCM. The study included 493 patients with median follow-up of 3.4 years. Their study showed that LV_LGE_ identified and reclassified certain population underestimated with conventional risk scores [34].

**Impact of LV_WT_, LV_MI_ and myocardial fibrosis on cardiac events and prevention of SCD**

Short-term and long-term studies have identified LV_WT_ as an independent predictor for VA [4,35,36]. However, the value of LV_WT_ to predict outcomes in patients with HCM is limited, with literature controversy. LVM offers more reliable representation of total LV hypertrophy compared to single wall thickness measurement, as shown in our study. Several studies highlighted the value of LV_MI_ as an independent predictor for SCD. CMR is more accurate in the assessment of LV_MI_ [28,32,33].

A recent retrospective study of 187 HCM patients by Dohy et al., 2021, demonstrated that CMR-derived LV_MI_ is an independent predictor for a major event and myocardial fibrosis (LV_LGE_) is a significant predictor for arrhythmia. Their patients were followed for an intermediate term (3.8 ± 2.4 years). The arrhythmia endpoint included malignant ventricular arrhythmia and appropriate ICD therapy. The incidence of death from all causes during follow-up was 10.7% (20/187) of patients. It is noted that their study population was younger (46.6 + 18.4 years) compared to our study (56.2 ± 16.3 years). Patients with ventricular arrhythmias had greater LV_MI_ of 126.2 ± 56.5 g/m^2^ and greater percentage of myocardial fibrosis of 13.1 ± 8.7% [33,37].

Myocardial fibrosis is another independent predictor for ventricular arrythmias, as shown in our study. Electrophysiological study of CMR LV_LGE_ territories revealed a significant correlation between myocardial fibrosis and the abnormalities of catheter-mapped electrophysiological parameters in relation to the occurrence of malignant ventricular arrythmias. Ventricular arrythmia could be linked to conduction block created by myocardial fibrosis, and re-entry circuit created by residual non fibrotic myocardium [15,38,39,40,41], with greater incidence of NSVT and ventricular ectopic with LV_LGE_. The risk of SCD is linearly related to LV_LGE_. Myocardial fibrosis > 15% was associated with a two-fold increased risk of SCD [16].

## 8. Implications of LV Mass/Fibrosis on Future Research in Hypertrophic Cardiomyopathy

There is a great interest in CMR-derived markers for risk assessment of patients with HCM. Our long-term study displayed significant association between LV_MI_ and LV_LGE_ and incidence of ventricular arrhythmias. Further investigations are required to assess the utility of adding CMR-derived markers for risk stratification of patients with HCM and improve identification of patients with HCM requiring ICDs for SCD prevention [42]. Myocardial biochemical changes have been demonstrated in some studies such as copper hemostasis.

Trientine demonstrated increased urinary copper excretion, with improvement in cardiac strain function along with a reduction in LV mass in this population [43]. LV_LGE_ has limitations with sequences, heart rate and kidney disease. Hence, LV_MI_ might be a more suitable, reproducible option without added software renderings and post-processing that is required for LV_LGE_ for risk stratification. LV_MI_ can be researched by conducting a randomized control study allocating moderate-risk patients with LV_MI_ > 85 g/m^2^ to either receive an ICD or a long-term continuous monitoring device such as an implantable loop recorder (ILR) for continuous risk assessment looking for NSVT as opposed to using extended ambulatory monitoring with poor diagnostic yields.

## 9. Limitations

There were limitations in our study related to retrospective data acquisition. The capture of VA using Holter monitoring is likely to result in underestimation compared to ICD monitoring, and the use of implantable loop recorders would give a more accurate capture of VA. Another limitation was the use of a combined endpoint e.g., NSVT, ATP and ICD shock. The last major limitation is that non-sustained VT does not necessarily translate to SCD over five- to ten-year follow-up, although this has not been extensively studied.

## 10. Conclusions

LV_MI_ and myocardial fibrosis are strongly associated with ventricular arrhythmias over long-term follow-up of HCM patients. The utility of these CMR markers as risk stratification tools needs to be further investigated in a randomized control study.

## Figures and Tables

**Table 1:** Demographics of patients with hypertrophic cardiomyopathy confirmed by MRI.

Parameter		All N = 252 Mean ± SD (95% CI)	Group A VT +ve N = 68 Mean ± SD (95% CI)	Group B VT −ve N = 184 Mean ± SD (95% CI)	*p* Value
**Age of diagnosis (mean ± SD)**		56.2 ± 16.3 (54.2–58.2)	53.9 ± 17.4 (49.3–57.7)	57.2 ± 15.8 (54.8–59.5)	0.18
**Follow-up years (median, IQR)**		5.9, (3.6–9.2)	7.7, (4.3–10.9)	5.5 (3.5–8.5)	0.01
**Male, n (%)**		174 (69.0)	49 (72.1)	125 (67.9)	0.33
**SCD family history, n (%)**		64 (25.9)	16 (23.5)	48 (26.1)	0.41
**ICD insertion < 2014**		19	16(25.4)	3 (1.6)	0.03
**ICD insertion > 2014**		16	8 (12.7)	8 (4.4)	0.04
**Holter monitor captured arrhythmia, n (%)**		39 (15.9)	39 (61.9)	0	n/a
**Death—all causes, n (%)**		29 (11.5)	11(16.2)	18 (9.8)	0.18
**Aborted SCD ICD/external defibrillator**		7	5/2	0	<0.001
**TTE**	LV_WT_ (mm), mean ± SD (95% CI)	16.7 ± 5.6 (16.0–17.4)	17.5 ± 6.2 (15.9–19.0)	16.4 ± 5.4 (15.6–17.2)	0.18
	LVOT gradient (mmHg), mean ± SD (95% CI)	9.5 ± 18.7 (7.2–11.8)	9.9 ± 22.9 (4.4–15.5)	9.3 ± 17.0 (6.9–11.8)	0.83
	LA diameter (mm), mean ± SD (95% CI)	39.3 ± 8.8 (38.2–40.4)	39.0 ± 9.1 (36.8–41.2)	39.4 ± 8.7 (38.2–40.7)	0.75
	ESC risk score, mean ± SD (95% CI)	2.15 ± 1.97% (1.91–2.40)	3.65 ± 2.85 (2.96–4.34)	1.57 ± 1.04 (1.42–1.73)	<0.001
**CMR**	LVEF (%), mean ± SD (95% CI)	71.1 ± 10.0 (69.8–72.4)	68.8 ± 10.3 (66.2–71.5)	71.9 ± 9.8 (70.5–73.4)	0.04
	LVEDV (mL/m^2^) mean ± SD (95% CI)	72.0 ± 16.1 (69.9–74.1)	72.0 ± 17.1 (67.4–76.6)	72.0 ± 15.8 (69.7–74.4)	0.75
	LVESV (mL/m^2^) mean ± SD (95% CI)	21.6 ± 10.5 (20.2–23.0)	21.9 ± 12.1 (18.5 ± 25.3)	21.5 ± 9.9 (19.9–23.0)	0.83
	LAVI (mL/m^2^), mean ± SD (95% CI)	51.3 ± 26.2 (47.1–55.6)	50.9 ± 23.7 (43.1–58.7)	51.5 ± 27.1 (46.3–56.6)	0.91
	LA diameter (mm), mean ± SD (95% CI)	36.5 ± 9.1 (35.4–38.4)	36.1 ± 9.3 (33.7–38.4)	36.7 ± 9.0 (35.4–38.0)	0.65
	LV_WT_ (mm), mean ± SD (95% CI)	19.5 ± 5.2 (18.9–20.2)	20.9 ± 7.0 (19.2–22.7)	19.0 ± 4.4 (18.4–19.7)	0.01
	LV_MI_, g/m^2^ mean ± SD (95% CI)	81.8 ± 28.4 (78.3–85.4)	90.3 ± 27.3 (83.7–96.9)	78.7 ± 28.3 (74.6–82.8)	0.004
	LGE, g/m^2^ mean ± SD (95% CI)	5.5 ± 5.4 (4.6–6.4)	7.3 ± 6.3 (5.7–8.9)	4.7 ± 4.3 (3.5–5.3)	0.01
	LGE %, mean ± SD (95% CI)	7.1 ± 6.6 (6.0–8.2)	9.2 ± 7.9 (6.7–11.8)	6.2 ± 5.9 (5.1–7.4)	0.02
	ESC risk score, mean ± SD (95% CI)	2.37 ± 1.95 (2.13–2.62)	3.95 ± 2.78 (3.27–4.62)	1.77 ± 1.02 (1.62–1.92)	<0.001

Footnote 1: ESC—European Society of Cardiology, g/m^2^—grams per meter squared indexed to body surface area, IQR—interquartile range, LA—left atrium, LAVI—left atrium maximum indexed volume, LV—left ventricle, LVOT—left ventricular outflow tract, LV_WT_—left ventricle maximum wall thickness, SCD—sudden cardiac death, SD—standard deviation.

**Table 2:** Univariate and multivariate logistic regression analysis for variables to predict ventricular arrhythmia.

Parameters	Cox Regression Analysis		
	**HR**	**95% CI**	***p* Value**
**Univariable**			
Age	0.99	0.97–1.01	0.16
Family history	0.87	0.46–1.67	0.68
LA diameter _(TTE)_	0.99	0.96–1.03	0.75
LVOT gradient	1.01	0.99–1.02	0.83
TTE LV_WT_	1.03	0.99–1.09	0.18
CMR LV_WT_ *	1.07	1.02–1.13	0.01
CMR LV_MI_	1.01	1.004–1.02	0.006
LV_LGE_	1.07	1.01–1.13	0.03
**Multivariable**			
CMR LV_MI_	1.02	1.01–1.04	0.003
LV_LGE_	1.08	1.02–1.14	0.02

Footnote 2: CI—confidence interval, LA—left atrial, LVOT—left ventricular outflow tract, LV_WT_—left ventricular wall thickness, LV_MI_—left ventricular mass index, HR—hazard ratio; * LV_WT_ was removed from multivariable analysis due to collinearity with LV_LGE_ and LV_MI_.

## Data Availability

Not applicable.

## List of Abbreviations

ACC	American College of Cardiology
AHA	American Heart Association
ATP	anti-tachycardia pacing
CI	confidence interval
CMR	cardiac magnetic resonance imaging
ECG	electrocardiogram
ESC	European Society of Cardiology
HR	hazard ratio
HCM	hypertrophic cardiomyopathy
ICD	implantable cardioverter defibrillator
LGE	late gadolinium enhancement
LV	Left ventricle
LV_LGE_	left ventricle late gadolinium enhancement
LV_MI_	Left ventricle mass indexed to body surface area
LVOT	left ventricle outflow tract
LV_WT_	left ventricle wall thickness
NPV	negative predictive value
NSVT	non-sustained ventricular tachycardia
PPV	positive predictive value
ROC	receiver operative characteristic
SCA	sudden cardiac arrest
SCD	sudden cardiac death
SD	standard deviation
TTE	transthoracic echocardiogram
VA	ventricular arrythmia
VT	ventricular tachycardia
VF	ventricular fibrillation
HRK	Conceptualization: data gathering, data analysis, drafting of manuscript, and editing.
PR	Data gathering, editing and proofreading of manuscript.
AHT	Data gathering, MRI analysis, drafting of manuscript, editing and proofreading.
MA	MRI analysis, editing manuscript and proofreading.
APT	Editing manuscript and proofreading.
KS	Data gathering, editing and proofreading of manuscript.
AAA	Data gathering, editing and proofreading of manuscript.
BE	Editing manuscript and proofreading.
AU	Editing manuscript and proofreading.
TM	Conceptualization, editing and proofreading.
AG	Editing manuscript and proofreading.
